# The art of friendliness: Organiser perspectives on curating dementia friendly cultural events

**DOI:** 10.1177/14713012231158429

**Published:** 2023-02-17

**Authors:** James Rupert Fletcher, Maohui Deng, David Dobson

**Affiliations:** Department of Sociology, 5292 University of Manchester, UK; Department of Drama and Film, 5292 University of Manchester, UK; Department of Sociology, 5292 University of Manchester, UK

**Keywords:** arts, performance, social prescribing, culture, friendliness, dementia friends

## Abstract

Over recent decades, the arts have become a popular response to dementia. Amidst wider concerns with accessibility, widening participation and audience diversity, coupled with greater attention to creativity across dementia studies, many arts organisations are now offering dementia friendly initiatives. While dementia friendliness has been well-established for almost a decade, the meaning of friendliness remains vague. This paper reports results from a study of how stakeholders navigate this nebulousness when developing their own dementia friendly cultural events. To assess this, we interviewed stakeholders working for arts organisations in the northwest of England. We found that participants built up local informal networks of knowledge exchange, sharing experiences between stakeholders. The dementia friendliness that characterises this network centres on the crafting of vibes that enable people with dementia to ‘unhide’ themselves. Through this accommodating approach, dementia friendliness converges with stakeholder interests, becoming something of an art form in its own right, typified by active embodied experience, flexible and creative self-expression, and being in-the-moment.

## Introduction

Since the 2000s, various cultural initiatives have been championed as therapeutic responses to dementia (Zeilig et al., 2014). We use “cultural” here in an expansive sense, referring to initiatives designed and orchestrated by cultural institutions and professionals, encompassing spirituality (Ennis & Kazer, 2013), theatre (Dassa & Harel, 2019), cinema (Brittain et al, 2017), dance (Mabire et al., 2019), music (Dowlen et al, 2018), literature (Billington et al, 2013), visual art (Shoesmith et al., 2021), textiles (Kenning & Treadaway, 2017), games (Zheng et al., 2017), gardening (Smith-Carrier et al, 2021) and more. While evidently distinct in some ways, all these forms of cultural activity have been promoted as dementia interventions in a similar manner over recent years. It is therefore analytically useful to collate them as a category of social phenomena. These initiatives are united by a shared philosophy of realising some instrumental potential of the creative sector as a medium of cognitive therapy and/or care provision.

The turn to cultural intervention during the 21^st^ century sits at the intersection of several social, political and economic trends. For instance, the disappointing pharmacological landscape, replete with frequent drug discovery failures, has fuelled interest in developing non-pharmacological therapies. This has been aided by the emergence of dementia studies as a significant sub-specialty in the humanities since the 1990s. At the same time, challenging fiscal circumstances in the creative sector have inspired rearticulations of cultural activities as health interventions, emphasising their instrumental value to justify financial support (Zeilig et al., 2014). The positioning of culture as therapy has also coincided with the growing popularity of social prescribing in England, which was enshrined in the NHS Long Term Plan (NHS, 2019). Healthcare practitioners are encouraged to direct isolated patients with chronic conditions toward various social initiatives outside of traditional healthcare services (Drinkwater et al., 2019).

Intersecting with the cultural turn in dementia intervention, the concept of “dementia friendliness” has similarly been popularised in the early 21^st^ century, both in the UK and internationally. Dementia friendly initiatives typically focus on environments and/or awareness, based on the rationale that places and people can be adapted to become more conducive to wellbeing in dementia (Hebert & Scales, 2019). The spread of dementia friendliness was one of the central aims of the Prime Minister’s Challenge on Dementia in 2012 and has subsequently been pursued by the Alzheimer’s Society through its dedicated Dementia Friends programme, which has recruited millions of “Friends” alongside businesses and celebrities (Parkin & Baker, 2021). The contemporary popularity of friendliness is entwined with that of cultural interventions. When the Alzheimer’s Society published its implementation guide on friendliness in 2015, art and culture was the first priority area identified (Turner & Cannon, 2018). This national effort is echoed in guidelines published by international organisations such as the World Health Organisation, which recently published a toolkit for developing dementia friendly initiatives, including advice for arts-based and cultural enterprises (WHO, 2021). Hence, the intersection of cultural interventions and friendliness is a truly global affair, with examples in the US (Harris & Caporella, 2019), Canada (Humphrey et al, 2019), Australia (Dionigi et al, 2018) and the Netherlands (van de Ree, 2018).

Though often well-intentioned and widely celebrated, dementia friendliness remains vague and heterogeneous, both in policy and practice (Brittain & Degnen, 2022; Rahman & Swaffer, 2018). The British Standards Institute published a voluntary code of practice in 2015, but it is remarkably vague and is rarely referenced in the literature. It notes that friendliness “aims to help people living with dementia to feel included in their local communities, with choice and control over their day-to-day lives”, which is achieved through raising public awareness and making architectural modifications (BSI, 2015). The Alzheimer’s Society continues to signpost the BSI code via its Dementia Friends platform, which defines friendliness as “improving inclusion and quality of life for people with dementia in a number of ways” (Dementia Friends, 2017).

Definitional expansiveness creates significant space for stakeholders to tailor friendliness to their own needs, which can be helpful. Unfortunately, ill-considered and formulaic approaches risk worsening the circumstances of people affected by dementia by othering them, with “friendliness” too often serving the interests of the organisations promoting it (Fletcher, 2021; Fletcher & Maddock, 2021; Swaffer 2014). This is not to suggest that friendliness should be systematised into a more tightly defined model, which could prove difficult to reconcile with diverse contexts and personal preferences. Instead, it serves to reveal that one cannot simply learn dementia friendliness and then put it into practice in an unproblematically straightforward manner, which presents a challenge for those seeking to develop friendly initiatives.

Some have welcomed the focus on friendliness and culture as representing moves away from a (bio)medical model of dementia, replacing individual “loss and tragedy” with “well-being, human rights, and social inclusion” (Hebert & Scales, 2019: 1859). Others interpret these trends as a liberal and austere reimagining of care, replacing costly care services with piecemeal community initiatives, de-professionalising and de-valuing the arts, artists, care and carers. Appeals to friendliness and cultural intervention can hence manifest an instrumental politics of public health based on behaviour management, cost-cutting and community responsibilisation (Bamford & Berry, 2012; Lotherington, 2019). Ultimately, despite increased popularity, the politics of dementia friendly cultural events remain as contested as their conceptualisation and implementation, posing further difficulties for those seeking to organise events.

It is important to specify that this paper does not offer an outcome evaluation. Much of the literature on friendly and cultural intervention focusses on such evaluation, resulting in an uncertain evidence base indicating potentially positive, albeit vague, effects. The robustness of evaluation is repeatedly debated, with little progress (Gray et al, 2018; Hebert & Scales, 2019). Gray et al. (2018) have suggested that the underlying idea of cause-and-effect outcome measurement is methodologically and ethically ill-suited to arts-based dementia interventions, which perhaps explains the impasse. Rather than adding to the collection of intervention evaluations and associated debate, in this study we explore how organisers conceptually and practically navigate the politics, promise and nebulousness of appeals to dementia friendly cultural events when developing their own initiatives. As such, rather than outcomes and theories, this study aims to provide a more pragmatic conceptual analysis of dementia friendly cultural events as they are envisaged and enacted in practice.

## Methods

We sought to answer the question: How do arts organisations and practitioners make their events dementia friendly? Our core interests concerned how stakeholders negotiate the conceptual and practical uncertainties that characterise the contemporary popularity of dementia friendliness and cultural interventions. To gain insight, we interviewed key stakeholders working for arts organisations in the northwest of England that either had experience of, or were in the process of organising, dementia friendly cultural events. For the purposes of sampling, stakeholders are defined expansively as people working in a formal capacity for organisations that are primarily dedicated to developing, supporting or implementing arts and/or cultural events and initiatives.

To access participants, we drew up an initial purposive sample of 31 suitable organisations and practitioners based on the authors’ significant collective experience of the regional cultural sector. We emailed each of these with an invitation to participate, an information sheet and a request to pass on the information to any other organisations who satisfied the study criteria. This resulted in a final sample of 11 participants from 10 organisations. Given the tight overall sampling frame, we have withheld further details about participating organisations to protect confidentiality. Each recruited participant was interviewed one time via Zoom. On average, interviews lasted around 1 hour. The interviews were semi-structured, based on a topic guide (appended) that was split into 2 sections, the first attending to participants’ perspectives on the conceptualisation of dementia friendly cultural events and the second focussing on their experiences of orchestrating such events.

The interviews were automatically recorded and transcribed using MS Word, and the resulting transcripts were edited to remove mistakes. Transcripts were analysed separately by two researchers using reflexive thematic analysis (RTA). This is a subtype of thematic analysis that pursues the same general aim of patten recognition in datasets, while also respecting the subjectivity of data interpretation. We chose RTA as a “big Q” qualitative paradigmatic technique that is conducive to a critical realist stance regarding the nature of and relations between data and meaning, befitting the study’s ontological, epistemological and methodological parameters (Braun & Clarke, 2021).

For each researcher, the analysis process began with familiarisation by reading the transcripts and taking brief notes. Each researcher then coded the transcripts, re-reading through each and summarising the meaning of passages in a word or phrase that captured the meaning. Using these codes, the researchers then separately generated initial themes by collating codes. At this point, the researchers met to review and develop their initial themes, each explaining to the other how they had interpreted the meaning of the data and comparing seemingly important codes. Somewhat surprisingly given RTA’s centring of subjective interpretation, the researchers generated several highly similar themes, albeit via distinct coding. Importantly, in line with RTA, we do not suggest that consensus is necessarily an indication of quality (Braun & Clarke, 2021). In discussion, the researchers refined their themes in reference to one another’s (theme framework appended).

The resulting 3 themes are presented below. These are *Networked Knowledges*, *Unhiding Vibes* and *Artful Friendliness*. To protect participant identities and clarify who is speaking, we consistently refer to them throughout this paper via their roles, without reference to their employing organisations. The project was funded by a Research Recovery Fund grant from the Faculty of Humanities at the University of Manchester and received ethical approval from the University of Manchester Research Ethics Committee (2022-13681-22437).

## Findings

### Networked knowledges

All participants emphasised the importance of knowledge sharing when developing dementia friendly events. In practice, participants engaged in such exchange via a web of associated actors, much of which was informal. This knowledge network included other cultural organisations and particular employees thereof, health and social care providers, universities, specific academics, community groups, local government and charities. Several participants went as far as contending that friendliness itself is “about making sure that you are working with the right partners” [arts centre director]. This networking of knowledge is informal and seemingly fragmentary, often relying on personal relationships. Indeed, several participants advised us to speak to specific people at other organisations. With this in mind, an arts charity director described their role as being:

Very much about collaborating, connecting, bringing people together, uniting people around a common message really. What we feel we've got a role to play in doing is trying to take a very fragmented ecosystem around music and dementia and make It more coherent.

Knowledge networks were typically local. Several participants noted that the Northwest has become a particularly fertile area for dementia friendly cultural events over recent years due to interest from local government, universities and cultural organisations. However, there were also national influences. Predictably, the most consistently mentioned of these was the Alzheimer’s Society Dementia Friend scheme, which was mentioned in some form by half of our participants. Some fully advocated this scheme:

I already had some personal experience with it, so understood a little bit, but there was so much that I didn't know. So when I got this job, I did a lot of research and have done some training with Alzheimer's Society. I’m trying very hard to get everyone in my organisation to become a dementia friend [theatre producer].

However, most participants were wary of an over-reliance on Dementia Friends as a guide for developing friendly initiatives and typically rejected formal training in favour of peer exchange and trial and error. In particular, they noted the danger that formal sessions on friendliness allowed stakeholders to claim that they are dementia friendly simply by virtue of having participated, without developing more substantial practice:

So many places you speak to and they go, “Oh yeah, we’re dementia friendly ‘cause all our staff have done dementia, friends training” and and you know, And the reality is that I suppose it's about much more than that really [theatre practitioner].

While the role of the Alzheimer’s Society was contentious, there was an almost ubiquitous view that carers were an invaluable source of knowledge on what constitutes dementia friendliness. To this end, several participants lavished praise on the wisdom of carers:

We learn from carers all the time. Their approach is masterful a lot of the time [theatre practitioner].

You get to know more about dementia through the carers, ‘cause they are the ones experiencing the full Dimensions of all the intricacies of dementia [musician and producer].

These appeals to the invaluable first-hand experience that carers could provide were generally not emulated in a similar position toward people with dementia. Most participants had personal experience of family members with dementia and claimed that this informed their work. However, dedicated engagement with people with dementia when developing initiatives was limited. Recognising this tension between consulting carers and people with dementia, a musician and producer reflected on who the dementia friendly cultural agenda was really for:

I know what child friendly is. You know things like stair gates, putting cupboard latches on and things like that. It means child-proof, but dementia friendly – does that mean dementia-proof? There’s a housing scheme that they’re setting up specifically for people with dementia, and to stop people with dementia walking off at night, they painted on the doorsteps a black hole to stop wandering, which I suppose is a bit like dementia-proof. It’s sort of prevention. Who is it aimed at? Child friendly, is it aimed at the parents or the child? Is dementia friendly aimed at the carer or the person with dementia?

This tension speaks to the wider relational stakes underpinning friendliness, which we return to below. It is important to note that, while in the minority, some participating institutions did proactively seek and respond to instruction from people with dementia. One organisation asked a person with dementia to visit the venue and conduct an audit, which was subsequently used to make a series of physical adaptations. An arts charity director went as far as to claim that the lack of consultation of people with dementia was fundamentally antithetical to the development of friendliness:

What’s not dementia friendly is when they’re [people with dementia]. . . not consulted with, and it's all very much done to them rather than with them.

Overall, the tying down of what dementia friendliness is and how it should be done was reliant on a large but piecemeal network of local stakeholders, characterised by informal relationships. Indeed, at the end of the interviews several participants stated that one of the motivations for being involved in this study was the potential for networking with local stakeholders and thereby further developing their own programmes. Participants articulated an ambivalent relationship with the national Dementia Friends scheme – which one might expect to be more influential given its profile – an enthusiastic commitment to carers as sources of knowledge, and a surprising distance from people with dementia themselves.

### Unhiding vibes

The dementia friendliness that emerged from the networking of knowledge can be characterised as revolving around the curation of unhiding vibes. By this, we mean that friendliness was often described as a somewhat ineffable vibe-like quality of the overall experience, with a particular quality that enlivened people with dementia by challenging the obscuring effects of dementia, both by virtue of the syndrome and other people’s responses, and bringing them into relational human existence. A musician and producer articulated this unhiding:

Making something a good place for people with dementia would be to not hide people away. It’s exposure. These are real people, you know. Don’t hide them away. It's dispelling the myths of people with dementia like they just wet themselves and they’re just dribbling in a corner. It’s like I said, you can’t tell who has dementia. Sometimes, I couldn't tell who's a person with dementia or the family member. It’s giving people opportunity to unhide themselves.

When discussing what friendliness was, most participants began by claiming a lack of expertise or clear knowledge, but would then progress to outline detailed and consistent accounts of friendliness, sharing several key features. The first of these was a commitment to being in the moment:

In terms of the content of the activity, it will be making sure that it’s very sort of in the moment and present [theatre director].

We really look at imagination and participation, so the value of in the moment participation for people living with dementia [theatre practitioner].

A musician and producer cited Dowlen et al., (2018) work on “in the moment” framings of music initiatives for people with dementia, echoing the trend described above for universities and even specific academics to be repeatedly invoked by participants. The importance of being in the moment was articulated as fostering a de-essentialising mundanity wherein all the baggage of dementia is removed. In the immediacy of crafting, singing or clapping, people are simply people. This artistic approach to humanisation resonated with a general conviction that people are unique individuals and should be respected as such. Several participants noted the importance of recognising that: “each person with dementia is so unique” [theatre director]. This was typically realised via commitments to personalisation. For instance, most participants extolled the virtues of familiarisation as a means of getting to know people with dementia and facilitating the tailoring of initiatives to them:

I think that's what our musicians do so well. They make an effort to speak to every single person individually, learn their names, learn their condition, and treat everyone individually. So it’s not just one homogeneous mass of people who’ve got dementia, it’s individual people with individual needs [musical project manager].

We really have the time over weeks to get to know them and know what sort of support they need. So I always plan the activities in mind of who is coming [music therapist].

While personalisation through familiarity is intuitively appealing, and was advocated by most participants, it can be difficult to realise in practice for various reasons. First and foremost, familiarisation requires time and therefore resources, which are often not forthcoming in either the cultural or care sector:

I’ve gone up to [funders] and said we need funding for the café, and they go, “we can probably give you £1000” and that’s great, but that’s going to pay for. . . just one session, and we know through research that benefits are accrued over at least twenty-week periods. Well, I’ve got to fundraise for 20 weeks’ worth of activity, otherwise it's just not worth doing one off [musician and producer].

Personalisation can also be difficult to achieve in the context of group-based initiatives. Most participants were primarily involved in cultural activities that involved groups of people, big or small, at the same time. Fostering relations was repeatedly identified as a key facet of friendliness, but the group-centred nature of initiatives inevitably meant that not everybody could be satisfied all the time:

It's just thinking in a really holistic human way, but thinking quite laterally as well in terms of what’s working for someone over there. Not working for another. What's the middle ground? How do we make this work in a group? And obviously it's much easier when you’re working on a one-to-one basis. In a group there's a balancing act between meeting needs [arts charity director].

One of the ways that participants managed this problem was through non-specificity. A lack of specificity was a characteristic feature of dementia friendly cultural events that was consistently advocated by participants. This deliberate non-specificity was favoured for various reasons. It enabled participants to develop personalised activities in response to people in the moment. It also prevented any essentialisation of dementia and othering of those affected, which participants worried could be a potential consequence of a heavy focus on dementia friendliness. Ultimately, this lack of specificity was articulated as something of a guiding philosophy, representing an overarching disposition toward de-essentialised engagements with people with dementia:

It’s not really so much about the specific things you do. It’s your approach to it. So like building it into the ways that you work more than anything else. It’s the intention, I think, and the willingness to want to be dementia friendly and making it a conversation [theatre producer].

Perhaps the most interesting manifestation of the lack of specificity that typified participant accounts of dementia-friendliness was the significant conceptual overlap with relaxed performances generally, and especially those relating to children and people with autism. Two participants considered interest in dementia friendliness to have emerged out of the earlier development of such accessible performances, which has proliferated in several directions. This created uncertainties regarding the boundaries of accessibility and the generalisability of core ideas:

The history of relaxed performances was more about people who are neurodivergent. But now at a relaxed performance, you might get mothers and babies, which is interesting because that could be really stressful for a person with dementia if there’s a crying baby. But then do you have friendly performances for everyone? How does that work? Then there’s an argument, should all performances be relaxed? There’s a really strong argument that it’s just about inclusion [theatre director].

A music therapist went as far as suggesting that this overarching commitment to accessibility, emerging in relation to autism specifically and within which the dementia friendly agenda is now situated, is blurring with concerns regarding audience diversity more generally:

When relaxed, performances first started happening, they were very targeted. They were more about autism, and I think relaxed performances have become something else now. I think you start to bring in a really diverse mix of audiences.

While participants’ accounts focussed on the general vibe of friendly initiatives, there was a consistent recognition that friendliness was also a physical consideration. Indeed, this was often articulated as a binary of overarching approach and environmental adaptations. These physical adaptations included accessible toilets, clear signage, no exuberant patterns, soft focussed lighting, soft soundscapes and transport provision. Perhaps most notable was the removal of black mats, a feature of dementia friendliness expressed by most participants. We were naturally curious about the ubiquity of this rather specific characteristic, but we were unable to pinpoint it beyond a general awareness that seems to have spread throughout the local knowledge network. Overall, while recognising a need for physical adaption, most participants emphasised the greater importance of creating a positive vibe, ambiance, energy, etc. as a means of doing friendliness:

Rather than just being like “look, we’ve put up this nice yellow sign for you”, making it so that actually within the fabric of this place we’re trying really hard to include you and welcome you [theatre producer].

They [musicians] do it very much off the cuff. Whatever they think the vibe is, they'll go with it [musical project manager].

It's how you build a culture. How you build the culture that you want to cultivate and that you want to flourish [theatre practitioner].

It's the energy and the way you deliver it [music project facilitator].

The reasons behind the popularity of these vibe-centred conceptualisations of friendliness are not clear, but it was evident throughout the interviews that participants were committed to such an approach. Of course, this is not merely an intellectual commitment, but was based on years of collective practice. In participants’ experience, unhiding vibes work.

### Artful friendliness

As discussed, the popularisation of cultural events as a response to dementia has provoked criticisms of the instrumentalisation and exploitation of the arts and artists. With this in mind, the navigation of artistry and instrumentalism was discussed by all participants in different ways. Perhaps unsurprisingly, participants had a genuine belief that art could be therapeutic:

We know that music is that one thing that can cut through dementia regardless of the severity of it. We know that music, when it's applied in the right way at the right time by the right person, and it's the right music, has potential benefits that are unrivalled in comparison with other interventions [arts charity director].

While such therapeutic potential was widely acknowledged, there was also recognition, and in some cases resentment, that the development of the arts as a response to dementia was somewhat exploitative. One particular example of this that incensed several participants was the recent growth of social prescribing without corresponding funding allocations:

The things that should help us with this work like social prescribing actually don't because social prescribing assumes that the creative work is being delivered by volunteers. It doesn’t actually pay for artists to deliver creative works [theatre practitioner].

A musician and producer was particularly exasperated by this situation and the financial assumptions underpinning it:

One quite large bugbear is that we’re getting a lot of social prescribing referrals. We’re a charity and we have to fundraise for everything we do. . . Imagine if a doctor said: “I’ve written you a prescription for 4000 paracetamol” and I was like: “Ok I’ll just walk into Boots and take it off the shelf.” Boots has loads of money, but you still have to pay. No one is giving us money. They’re going through a charity that is relying on trusts and foundations to provide an amazing service for free, and that for me is wrong.

However, while there was significant concern regarding the financial circumstances of this situation and related potential for exploitation, wider concerns regarding instrumentalisation of the arts did not seem to be shared by participants. Indeed, the general sentiment was quite the opposite. It became clear that, for participants, the core principles of dementia friendliness and the arts are remarkably concordant. The in-the-moment focus, extolled by various participants, was a particularly good example of the potential for convergences between artistry and friendliness. Participants described cultural activities as temporally grounding those involved, often using active embodied experiences:

It’s like an anchor for people to guide themselves or be guided back to that moment. We call it participation by stealth, so that people are participating before they realise, so they were already halfway into a workshop before they even realise it started. That might be like putting on a piece of music or just starting something that's interesting, like handling something that people are curious and interested in [theatre practitioner].

As well as emphasising embodied experience and grounding people in-the-moment, the arts were also described by participants as being especially conducive to the flexibility, and by extension personalisation, that is so often advocated as dementia friendliness:

How do you mould and make your medium malleable to their needs? I mean music’s mercurial isn’t it? It moves around. You can shape it and make it whatever you want it to be [arts charity director].

It's not a performance, it’s participating in the music, and there are no wrong notes or anything. It’s almost completely and entirely improvisation. It’s always playing with the rules. I am not leading the entire session there. Roles are swapping and I give them the opportunity to be the leaders. So it’s lifting them up and creating situations where they can be the leaders and performers [music therapist].

Here, the music therapist notes the capacity for improvisation to develop that same unhiding-ness expressed by other participants, whereby people are almost unconsciously pulled into active forms of self-expression. There is a type of social model of disability in action here, through the curation of socio-political environments that are actively enabling. However, there is also no suggestion of an instrumentalisation or corruption of the arts. Ultimately, participants articulated dementia friendliness as an artfulness in its own right, furthering artistic concerns. For instance, a music project facilitator defined dementia friendliness as “a kind of joyous atmosphere that becomes very conducive to creativity.” A theatre producer explained how dementia friendliness resonated with the organisation’s commitment to creativity, challenging norms and fostering diverse participation:

We're a creative space. We have to deliver creative stuff. We can’t just do normal stuff all the time. That’s not what this organisation does. That’s why we want to kind of bring new people in, and we want them to encounter us.

Hence, dementia friendliness was not viewed by participants as something patched onto the arts. It was instead articulated as a medium of artistry, or even as a form of cultural activity in its own right (see also Saito, 2022; Thompson, 2022).

## Discussion

As noted, participants were generally restrained at the beginning of interviews, impressing that they were not experts on dementia or friendliness. However, they then quickly proceeded to develop sophisticated and consistent accounts of how they conceptualised and enacted dementia friendliness when curating their cultural initiatives. This consistency might be somewhat serendipitous, but it may well suggest that the knowledge networks within which these stakeholders participate are productive and effective, developing and disseminating particular forms of thought and corresponding practice. With this in mind, it is interesting that participants felt moved to begin their interviews by caveating their expertise. This cautiousness might be indicative of the nature of their knowledge, which is relatively resistant to formal definition, instead positioning friendliness as a sort of mutable ambience, with flexibility being one the most important characteristics. This is echoed in the informal nature of the knowledge networks, which perhaps also encourages those involved to be taciturn regarding their expertise in a manner that might be different were those networks to be formally recognised institutes offering accredited educational programmes.

Intuitively, one might be tempted to explore the prospects of formalising this knowledge networking into more generalisable and reliable resources. The network(s) described by participants were apparently piecemeal and highly dependent on individuals knowing individuals. This could present obvious barriers to involvement for outsiders. However, any formalising of this system might risk losing the fluidity and reliance on iterative peer experience that seems to characterise the evolution of knowledges in the dementia friendly cultural space. It certainly appeared that some participants were enjoying reasonable success by simply liaising with other stakeholders and developing initiatives through a sort of communal trial and error. Similarly, the local focus of the organisations seemed to help them tailor initiatives to specific audiences and foster familiarity. It could be that the flexibility of the knowledge and the generation of that knowledge are mutually reinforcing.

One area of knowledge exchange that might warrant further attention is the positioning of people with dementia. The relative lack of inclusion of people with dementia in the curation of what friendliness is and how it should be enacted is seemingly at odds with the explicit and often impassioned appeals to inclusivity that typified participant accounts. The simultaneous importance and lack of involvement of people with dementia has been similarly identified elsewhere (Darlington et al, 2021; Phillipson et al, 2019). This tension is further highlighted by the greater significance that many participants attributed to the knowledge of carers echoing longstanding tensions regarding the misuse of carers as accessible proxies for the experiences of people with dementia (Fletcher, 2020). While this relatively lesser inclusion was evident in processes of consultation and development, people with dementia were evidently highly involved in the curation of events through their active participant in those events. The inherent flexibility, responsiveness and commitment to personalisation meant that participants repeatedly outlined instances of adapting practices to suit people with dementia. One could reasonably argue that this does represent meaningful inclusion in the evolution of friendliness, perhaps even more so than the knowledge networking that precedes said events, but it was not articulated as such by participants. This potentially complexifies notions of consultation, inclusion and expertise when pursuing dementia friendliness.

Irrespective of how they are developed, the conceptualisations and practices of dementia friendliness that were articulated by participants were evidently well-formulated, and at the same time were somewhat removed from more official approaches. As noted in the introduction to this paper, dementia friendliness has been criticised for being overly vague (Brittain & Degnen, 2022; Rahman & Swaffer, 2018). However, it is often presented by organisations such as the British Standards Institute and the Alzheimer’s Society as revolving around awareness and community. Neither of these concerns featured in our participants’ accounts, clearly differentiating them from the wider literature’s focus on public awareness (e.g., Phillipson et al, 2019). The common focus on environments was evident, but the typical physical focus was consistently deemed less important than a more expansive atmospheric approach to creating friendly environments. Similarly, the five core messages of Dementia Friends did not feature prominently in any participant interviews and nobody mentioned the Alzheimer’s Society’s formal recognition process (see Dementia Friends, 2017). Instead, as presented, participant descriptions of dementia friendliness centred on the flexible curation of vibes that were attuned to the people involved, engaging them and encouraging them to be actively present. Dementia was depicted as a sort of blanket that smothered those affected, both through cognitive impairment itself and the reactions of other people to those affected, which cultural events had the power to remove, or to at least offer people with dementia some opportunity to partly escape from for an instant.

By partially distancing themselves from the official national channels of friendliness, e.g., the Dementia Friends scheme, Prime Minister’s Challenge on Dementia, etc. and instead developing more organic approaches, local cultural stakeholders can have considerable agency in determining the nature of dementia friendliness. Perhaps unsurprisingly, the result is a version (or versions) of friendliness that is remarkably concordant with their own interests and artistic sensibilities. There are at least substantive affinities between dementia friendliness and the doing of cultural events, and for many participants, it seems that the former is a version of the latter, troubling aforementioned critiques of instrumentalisation (Bamford & Berry, 2012; Lotherington, 2019). While participants regretted the financial implications of attempts to turn the arts into forms of therapy, there was no evidence to suggest that such approaches instrumentalised cultural events in a manner that corrupted the resulting artistry. In reconfiguring dementia friendliness as unhiding vibes revolving around in-the-moment non-specificity, flexibility and expression, cultural stakeholders were essentially realising friendliness as an art form.

The findings presented in this paper can be interpreted as offering some reason to be cautious about criticisms of dementia friendliness that claim it is problematically vague. In this study, that vagueness was evident but not necessarily problematic. Indeed, it facilitated (and perhaps even encouraged) iterations of friendliness that, from participants’ perspectives at least, had positive implications for both artistic stakeholders and people affected by dementia. This finding caveats critiques centring on a lack of outcome-based evidence (Gray et al, 2018; Hebert & Scales, 2019), suggesting that a tendency toward nebulousness might be a strength. This might also have repercussions for the development of relations between cultural organisations and care institutions as the arts are increasingly positioned as therapy, particularly through the growing popularity of social prescribing (Drinkwater et al., 2019). Participants welcomed the additional attention but regretted the lack of corresponding funding. However, one might reasonably expect that, were serious funding to be extended, then funders might impose more specific expectations regarding outcomes, audits, etc., which could be difficult to reconcile with the approaches to friendliness described by participants.

## Limitations

The major limitation of this study is the specificity of the sampling frame, being geographically limited to the Northwest of the UK. Participants remarked on the distinctiveness of local expertise in dementia-friendliness, so it could be that findings are removed from other areas of the UK. A larger national study could investigate this. Another limitation of the study could be the expansive operationalisation of cultural initiatives, which inevitably conflates various distinct institutions, practices and stakeholders. A larger study could investigate more particular differences within the cultural sector. Finally, as a stakeholder interview study, we can only offer participant reflections and are unable to report on experiences of the initiatives in practice. Similar future studies could incorporate participatory research in real-world events to broaden the scope of findings.

We hope that this paper can be, to some extent, a helpful resource for current and prospective stakeholders in the curation of dementia friendly cultural events. While we are cautious of encouraging a codification of things that currently seem to be meaningfully (if not crucially) uncodified, we also appreciate that it may be helpful for stakeholders to read about the distinct strategies of others with a view to emulating their successes and avoiding their mistakes. We hence recognise the potential paradox of extending phenomena such as “flexibility” as vital characteristics of friendliness. Ultimately, our results suggest a generally positive situation whereby regional commitments to dementia friendliness are being carefully and collaboratively pursued through various initiatives, albeit in the context of serious resource constraints. Perhaps most heartening is the manner in which, by practising with friendliness as something of an artform in its own right, stakeholders can curate strategies that are genuinely meaningful, engaging and rewarding for them and their organisations.

## Appendix 1. Topic guide

### Part 1 - Perspective on DF

• What is dementia?
• What is DF?
• What makes something un/friendly?
• Where do participant’s ideas come from?

### Part 2 Experience of DF

• Is the organisation interested in DF?
• What has been done? Why?
• Organised/contributed to/participated in specific events?
• What does/n’t work?
• Future plans?

## Appendix 2. Themes.

**Table:**

Unhiding vibes	Nonspecific	Access	
		Relaxed	
		Naming	
		Specific	
	Inclusion		
	Flexibility		
	Time		
	Awareness & stigma		
	Physical environment	Accessibility	Online
		Transport	
		Visual	
		Light	
		Sound	
		Sensory	
		Urbanicity	
	Vibe	Friendliness	
		Relaxed	
	People	Carers	
		Relational	Communication
		Friendly assistance	
		Friends training	
		Personalisation	Familiarity
		Person-centred	
	Humanisation		
	Presentism	Face-to-face	
	Clarity		
	Weird language		
	Simplified	Non-abstract	
	Perspective		
Networked knowledges	Dementia friends		
	Misc experts	International	
		Regional	
		Scholarship	
	Online		
	Carers		
	People with dementia		
	Experience		
Artful friendliness	Staffing	Contacts	
	Networking		
	Freedom		
	Motivations		
	Activities	Trial & error	
	Going to them		
	Instrumental	friendliness = art	
		Results	
	Examples	Social prescribing	
	History		
	Action		
Challenges	alzheimer’s society		
	Substituting care		
	Othering		
	Language		
	(dis)enfranchisement		
	Resources		
	Commitment		
	Technology		
	Immeasurable		
	Sceptics		
	Transport		
